# Modeling and Experimental Verification of the Performance of Polymer Composite Reinforcing Bars of Different Types in Concrete of Different Density

**DOI:** 10.3390/polym14091756

**Published:** 2022-04-26

**Authors:** Alexey N. Beskopylny, Sergey A. Stel’makh, Evgenii M. Shcherban’, Levon R. Mailyan, Besarion Meskhi, Innessa Efremenko, Valery Varavka, Nikita Beskopylny, Natal’ya Dotsenko

**Affiliations:** 1 Department of Transport Systems, Faculty of Roads and Transport Systems, Don State Technical University, Gagarin, 1, 344003 Rostov-on-Don, Russia; 2 Department of Engineering Geology, Bases, and Foundations, Don State Technical University, 344003 Rostov-on-Don, Russia; sergej.stelmax@mail.ru (S.A.S.); au-geen@mail.ru (E.M.S.); 3 Department of Roads, Don State Technical University, 344003 Rostov-on-Don, Russia; lrm@aaanet.ru; 4 Department of Life Safety and Environmental Protection, Faculty of Life Safety and Environmental Engineering, Don State Technical University, Gagarin, 1, 344003 Rostov-on-Don, Russia; reception@donstu.ru; 5 Project Management Center, Don State Technical University; Gagarin, 1, 344000 Rostov-on-Don, Russia; i.efremenko@sci.donstu.ru; 6 Research and Education Center “Materials”, Don State Technical University, Gagarin sq., 1, 344003 Rostov-on-Don, Russia; varavkavn@gmail.com; 7 Department Hardware and Software Engineering, Don State Technical University, 344003 Rostov-on-Don, Russia; beskna@yandex.ru; 8 Department of Technological Engineering and Expertise in the Construction Industry, Don State Technical University, 344003 Rostov-on-Don, Russia; natalya_1998_dotsenko@mail.ru

**Keywords:** steel reinforcement, polymer composite reinforcement, fiber reinforcement, nanomodified concrete, lightweight dispersion-reinforced concrete

## Abstract

Currently, there is a scientific and practical deficit in new methods of integrated technological and design solutions based on improving the properties of concrete as the primary material that perceives compressive loads, and its joint work with various types of reinforcing rods. A new system using an integrated engineering approach to the design of building structures is proposed, which involves minimizing their cost and weight through numerical simulations and an experimental verification of the operation of reinforcing bars made of various materials in concrete of various densities. The control of the bearing capacity of reinforced building structures on the example of compressed elements is proposed to be carried out using the developed recipe-technological methods at the manufacturing stage. The economic and environmental efficiency of nano modification with the help of production waste and the use of lightweight dispersion-reinforced concrete to obtain such structures was revealed. The most effective concrete formulations showed strength gains ranging from 10% to 34%. Ultimately, this led to an increase in the bearing capacity of the elements up to 30%. The application of such an integrated lean approach will allow saving up to 20% of resources during construction.

## 1. Introduction

Outdated technologies in the conditions of intensively growing volumes of buildings and structures being erected are a problem for modern construction, and they require improvement based on a scientific approach. For many decades, including the present, reinforced concrete has remained the main structural material for buildings and structures of various levels of responsibility. Many attempts by modern scientists and engineers to improve concrete technology have been successful; however, until now, the issues of transition to a qualitatively new level of structural composites remain open.

Some of the most relevant areas in terms of the construction of buildings and structures made of reinforced concrete are the following:

- searching for new solutions regarding concrete, from which reinforced concrete structures are built, namely: recipe-technological solutions that meet the latest science and technology criteria;
- constructive solutions comprising geometric shapes and reinforcing cages, which are the basis of reinforced concrete and an integral part for the perception of loads, including ultra-high ones;
- finally, engineering solutions related to the specific replacement and transition from traditional types of materials and structures to modified components, which are based on fundamentally new approaches in terms of technology, design, and calculations.

In this regard, an analysis of the currently existing solutions in terms of concrete science and the reinforcement of concrete structures is required, as well as an analysis of the types of concrete and reinforcement used in various products, and structures made of heavy concrete, including the possibilities of their combinations, layout, and collaboration.

An application of steel elements in concrete is considered in many works, among which [1,2,3,4,5,6,7,8,9,10,11,12] are highlighted. Concrete-filled rectangular steel tubes (CFRST) are currently popular in the construction of high-rise buildings and bridge structures. The study [2] analyzed the behavior of CFRSTs with different central angles under eccentric compression. A reasonable simplified approach to the calculation of the ultimate eccentric bearing capacity CFRST [2] is presented and confirmed. The influence of steel strength class, steel coefficient, types of clamps, and flexibility coefficient on the bearing capacity of such elements was studied in [3]. It was proven that the use of high strength steel and the improvement of the steel ratio can greatly improve the bearing capacity of SRC columns. A modified formula was proposed for calculating the bearing capacity of SRC columns, based on the limiting effect of stirrups and steel on concrete [3]. Steel fibers are used to improve the plasticity of high-strength lightweight [1] and ultra-high-performance fiber-reinforced concrete [9]. Steel fibers in the amount of 0–1.5% (by volume) were used in the concrete mix. High-strength fibrous lightweight concrete can perform better than ordinary concrete under extreme loads because it exhibited a much higher ductility [1]. A mixture with a large volume (6%), and a combination of two segments of steel fibers (3% each), a ratio of water and binder of 0.16%, and a ratio of superplasticizer and binder of 6.1%, showed the highest strength and deformability in the plastic region [9]. Reinforced concrete beams made of a fine-grained fiber composite with the addition of steel fibers in an amount of 1.2% by volume of the composite, using secondary filler, were studied for shear strength in terms of bending under the action of a transverse force, as well as cracking forces that cause the appearance of the first diagonal crack. The proposed new fibrous composite with fine aggregates can, in some cases, serve as an alternative to conventional concrete [10].

The behavior of Ultra High-Performance Concrete (UHPC) beams was studied using a finite element model that takes into account the response of UHPC and rebar to tensile and compressive stress-strain, the bond between concrete and reinforcing steel, and the effects of work hardening in bars and UHPC. The proposed model was claimed for application in the field of quantifying the contribution of stirrups and concrete to the shear strength of beams to study the possibility of removing transverse reinforcement in UHPC beams [13].

Hybrid fiber reinforced concrete, reinforced with two types of fibers (steel and polyvinyl alcohol), was studied to create a stress-strain model to predict the strength of steel fiber concrete in the range from 40 to 120 MPa. It was shown that an increase in the content of steel fiber to a certain level, at a constant amount of fiber from polyvinyl alcohol, leads to an increase in stress resistance values [14].

The effect of adding different types of fibers to concrete mixtures on the shear behavior of two-span fiber-reinforced concrete beams with or without shear reinforcement was studied by the authors in [15]. Steel or basalt fiber was used with dosages of 78.5 and 5 kg/m^3^. It is summarized that the shear capacity increased in concrete beams with steel or basalt fibers. In this case, the type of fracture changed from shear (brittle) to flexural shear (less brittle) [15]. Increasing the bearing capacity of reinforced concrete structures, reducing the consumption of materials, and ensuring quality, is possible using steel rods of increased strength and various types of fibers [16,17]. Research into High Performance Fiber Reinforced Concrete (HPFRC) is quite common at the present time. Much attention is paid to factors such as the dosage of fiber in the composition of concrete, as well as its surface and length, which have the greatest impact on the structure of fresh and hardened concrete, as well as its mechanical properties [18,19].

The works [20,21] contributed hugely to the development of the study of fibrous porous media. The proposed fractal models successfully described the dependences of microstructural and electrokinetic parameters of porous media [20], permeability, and the Kozeny–Carman constant [21] on porosity, equivalent particle diameter, ratio of the minimum pore radius to the maximum pore radius, molar concentration, zeta potential, fractal dimension tortuosity, and fiber diameter [20,21].

Fiber-reinforced concrete is widely used to increase the durability of reinforced structures. The redistribution behavior of bending and shear moments in fiberglass-reinforced and polymer-reinforced (GFRP) continuous concrete beams was studied in [22]. The paper [23] presents an estimate of the bending strength of concrete with the addition of 2.0 and 3.0 kg/m^3^ of synthetic fibers of various geometries and shapes. An increase in bending strength up to 13.5%, depending on the type of mixture, and an increase in plasticity were noted. It is summarized that the proposed fiber-reinforced concrete mixtures cannot replace traditional reinforcement as steel rods [23].

Modeling and calculation of hollow reinforced concrete elements were considered in [24,25]. The reinforcement of hollow concrete cylinders with nickel–titanium alloy (Ni–Ti) memory alloy (SMA) wires wound around them was considered using a model for analyzing thermal stresses in a concrete shell. Almost a year after prestressing with Ni–Ti SMA wire, it was confirmed that the residual stress in the wire is maintained and effective for a long time [24]. Simulation of the earthquake behavior of corroded reinforced concrete hollow poles by FB-FEM was used to study the effect at failure using collision calculations and stepwise dynamic analysis considering different corrosion rates [25,26].

Textile mortar reinforcement (TRM) of reinforced concrete (RC) columns through the shell, under combined axial and cyclic loads, showed that increasing the length of the shell improves the lateral deformation capacity, and linearly increases the length of the plastic hinge up to a constraint factor of 0.2. Mortars with higher flexural strength resulted in somewhat greater deformation capacity; however, the difference in the compressive strength of the solution did not affect the ultimate capacity for lateral deformation [27].

An increase in strength and deformation characteristics due to the use of polymer rods was considered in [6,7,28,29,30,31,32,33]. Replacing steel rods with polymer ones can significantly increase the ductility and strength of reinforced concrete columns. The results showed that carbon fiber rods increase the rigidity of the models, whereas steel rods increase the coefficient of energy absorption and ductility [28]. The effect of the joint work of polymer rods and glass fiber in the context of the relationship between the strength characteristics in bending and the density of concrete was studied in [29]. An increase in the experimental shear capacity of concrete beams by using a carbon fiber reinforced polymer with improved compressive strength was considered in [30].

The joint use of experimental and computational methods of high-strength concrete and mortar subjected to a compressive load was reflected in [34]. At a lower studied stress level, basalt coarse aggregate improved the fatigue characteristics of concrete. Signs of a negative effect were observed at a higher level of stress [34]. Analytical and numerical approaches to the calculation of elements of reinforced concrete structures with basalt fibers were considered based on [35]. It is possible to reduce environmental pollution by using sustainable materials to produce sustainable concrete. Such materials are reinforcing fibers (steel, polypropylene, carbon fiber), recycled materials (tire rubber, crushed glass, plastic, industrial waste), as well as organic and inorganic elements such as concrete aggregates and reinforcing elements. Some resistant materials added to cement can improve the compressive and bending strength of concrete elements [36,37,38]. Compressed and bendable concrete elements with bounding reinforcement grids can be calculated according to a common method [39], considering all the main factors affecting the mechanical properties of volumetrically compressed concrete [32,40].

Works that studied the modification of concretes with polymers in various dosages, with various sizes of large aggregates, at various sample testing temperatures [41], and calcium carbonate nanoparticles of a rationally selected dosage [42], were also aimed at improving the characteristics and structure of concretes.

Summarizing the results of the review and its analysis, it should be noted that the scientific deficit and the practical gap based on it is associated with the applied development of new methods of complex technological and design solutions. They are based, firstly, on improving the properties of concrete as the main material that perceives compression loads and its joint work with various types of reinforcing rods. Focusing on the main principle of design improvement, maximum reduction of the weight and cost of the structure, and at the same time increasing its manufacturability, there is a need to check the compatibility of specific types of concrete of certain classes, and specific types of reinforcing bars made of certain materials with certain specified characteristics.

The works studied during the literature review are relevant, but they need to develop the theoretical ideas and practical recommendations received by these authors. In the work of the considered authors, several scientific and applied problems are identified that should be solved. These problems stem from the poor adaptability of traditional building technologies for modern complicated construction conditions. In particular, these problems are concerned with the large weight of buildings, complex engineering geological conditions, and dense urban development. These problems can be effectively solved, and the data already obtained by the authors of the considered works should be developed in the direction of creating new lightweight types of structures with various types of reinforcement in various types of concrete. Such questions have not yet been investigated and are unresolved. Our study aims to address and answer these questions. After a theoretical review of experimental studies and analytical interpretation, the novelty of our work will be the developed integrated approach that will solve these problems.

The objectives of the study are:

- the formulation of a working hypothesis, development of an experiment plan, and based on a selection of the most significant factors that are based on the analysis, results that affect the bearing capacity of compressed reinforced concrete elements;
- conducting large-scale numerical experiments aimed at identifying bottlenecks and points to strengthen solutions in terms of technology and design;
- the determination of directions and vectors that can be verified by laboratory-physical experiments;
- setting up a physical experiment in a laboratory to confirm a hypothesis in terms of technology;
- carrying out a design check of the proposed technological solution and, on that basis, developing a design proposal with justification from the point of view of efficiency on operational and economic grounds.

The scientific novelty of the research is:

- from the point of view of theory, the study of compatibility and the development of existing ideas about such compatibility concerning various types of materials and their interaction from the point of view of the stress-strain state, and obtaining new knowledge about their joint work. Both stone materials (concretes), metals, and various kinds of composite materials are studied thusly, and the theory and empirical basis for the interactions of various materials in the body of a single composite under a load is developed;
- various kinds of experimental data obtained with the help of special calculation programs and numerical experiments were investigated and applied, as well as physically verified and tested;
- complex technological and design solutions are presented, combining simplified methods of technological design, applicable, first, for further scientific research in this direction, and second, for the engineering and construction industry in the construction of buildings and structures of a new type.

## 2. Materials and Methods

One of the most important parts of the proposed integrated approach is the choice of research methodology. It should be understood that we are dealing with an even greater synergistic effect, which, in our opinion, arises from a simultaneous careful analysis of material science and the design components of the study. In this regard, our methodology is divided into the choice of initial components, recipes and technologies for laboratory physical experiments, as well as the appropriate material, software base, and verified calculation methods from the point of view of numerical experiments; therefore, in the section “Materials and Methods”, it is necessary to provide materials for conducting laboratory physical experiments, and the subsections detailing the methods should not only indicate the methods of testing and research in the conditions of the laboratory of the planned materials, but also the method of conducting numerical experiments using modern software. All this will allow us to obtain the necessary synergistic effect, which takes into account two components: materials science and design.

### 2.1. Materials

Reinforced concrete columns with a cross section of 400 mm × 400 mm, and a length of 3000 mm, 6000 mm, and 9000 mm, made of class B30 concrete, were chosen as the basic object of study. The subject of the study was the characteristics of the columns depending on the changing technological and design factors.

A previously developed composition with micro silica nano-modifier was used and adopted as heavy concrete for the experiments. It allows, using industrial waste, as an additive to heavy concrete, to achieve, first, a denser packing of particles, improving the quality of the structure of concrete, and allowing this concrete to acquire improved mechanical structural characteristics with an increase in the bearing capacity of elements obtained from such a concrete [43,44].

**Figure 1:**
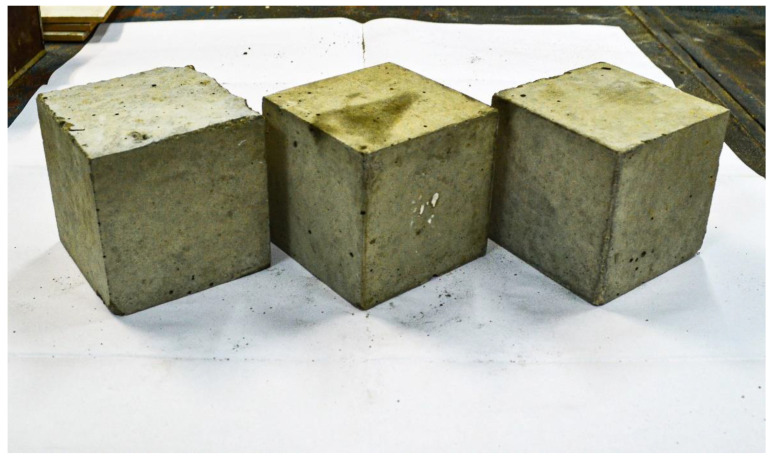
Samples of nano-modified micro silica heavy concrete for compression testing.

Samples of heavy concrete with nano-modified micro silica for compression testing are shown in Figure 1.

Information about the initial components used in the manufacturing of concrete using a nanomodifier is presented below.

When conducting research, we used Portland cement of the CEM 0 52.5N brand (Novoroscement, Novorossiysk, Russia), the physical and mechanical characteristics of which are presented in Table 1, the chemical composition—in Table 2, and the mineralogical composition—in Table 3.

Granite crushed stone (Pavlovsknerud JSC, Pavlovsk, Russia) was used as a large dense aggregate, and slag pumice (Stroymir LLC, Lipetsk, Russia) was used as a porous aggregate. The physical and mechanical characteristics of the aggregate are presented in Table 4.

Quartz sand (OOO Quartz Sands, Semenov, Russia) was used as a fine aggregate, the physical characteristics of which are presented in Table 5.

In numerical experiments, steel reinforcing bars (Tyazhpromarmatura, Aleksin, Russia), and polymer composite reinforcement (PCR) (Yaroslavl Composites Plant, Yaroslavl, Russia), were used as reinforcing elements. Characteristics of steel reinforcement are presented in Table 6, and polymer composites—in Table 7.

Micro silica grade MK-85 (OOO ZIPo, Lipetsk, Russia) was used as a nanomodifying additive in concrete. Table 8 shows the chemical composition of micro silica MK-85.

**Figure 2:**
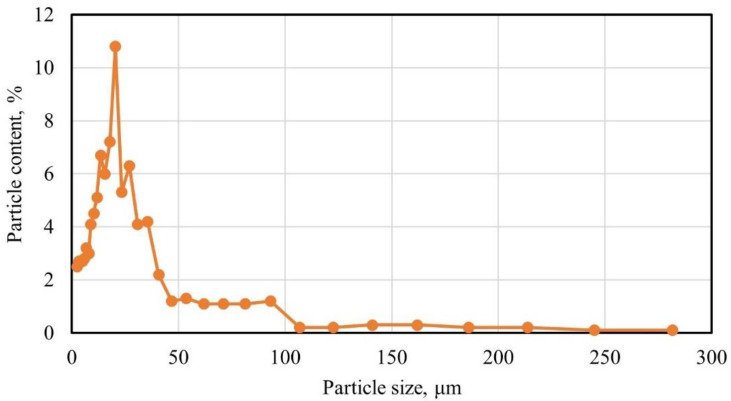
Granulometric composition of micro silica.

The granulometric composition of the applied nanomodifier is shown in Figure 2.

The micro silica particle size distribution plot shows that most of the particles (more than 80%) have a size of 2 to 40 µm, with the main peak at 20 µm.

**Figure 3:**
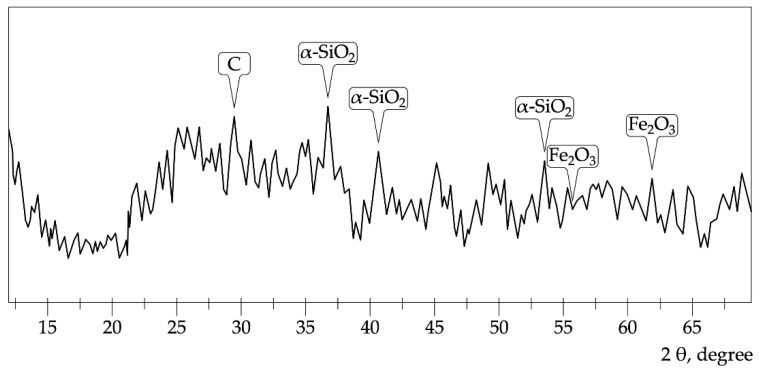
Micro silica diffraction pattern.

X-ray phase analysis of micro silica particles is shown in Figure 3.

Micro silica (Figure 2) is represented by amorphous silica, minor impurities of iron, carbonaceous substances, and crystalline α-quartz.

Lightweight fiber-reinforced concrete was also studied in accordance with [46,47,48,49,50]. Glass fiber (Armplast, Nizhny Novgorod, Russia) was used as dispersed reinforcing fibers, the physical and mechanical characteristics of which are presented in Table 9. Slag pumice (Stroymir LLC, Lipetsk, Russia) was used as a filler, the physical and mechanical characteristics of which are presented in Table 10. The mineralogical composition of slag pumice is characterized by light minerals (80%), which include calcium and magnesium carbonates (68%), quartz (12%), heavy minerals (18%), represented by onormanite in the form of short square prisms, and iron sulfate (2%).

### 2.2. Methods

The calculation was performed using SP 52-101-2003 [51], SP 63.13330.2018 [52] and LIRA-SAPR 2016 R5 software (Lira Service LLC, Moscow, Russia). This version of the software corresponds to the Standard plus configuration with the superelement mode, it has the ability to perform a full dynamic analysis, and the ability to check the strength of sections. It allows you to select sections of reinforced concrete elements using all standards.

The column was designed for central compression according to the formulas [51] p.6.2.17 for columns with a length of 3 m, 6 m, 9 m, concrete B30, B40, steel, and composite reinforcement. The accepted reinforcement is 4 longitudinal reinforcement bars with a diameter of 6 mm with a cross-sectional area of 1.13 × 10^−4^ m^2^.

**Figure 4:**
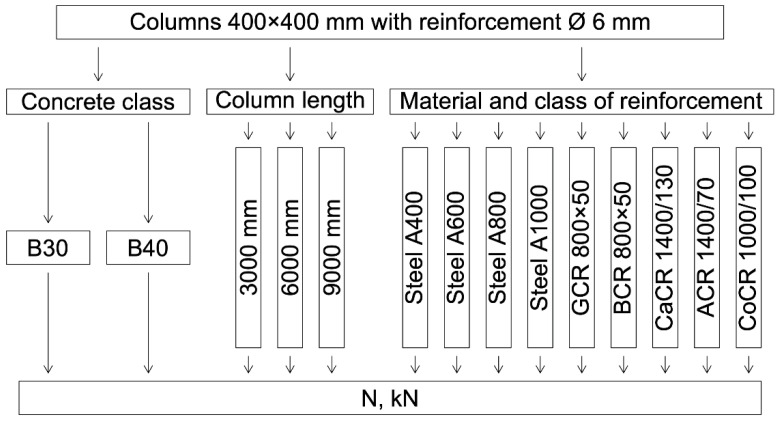
Plan of the numerical experiment.

The plan of the numerical experiment is shown in Figure 4.

According to [51], the limiting value of the longitudinal force (bearing capacity) was calculated:

(1) $$N_{ult}=\varphi \left(R_{b}A+R_{sc}A_{s.tot}\right)$$

where *R_b_* is the design resistance of concrete to axial compression for the limit states of the first group; 

*A* is the area of all concrete in cross section;

*R_sc_* is the resistance of reinforcement to compression;

*A_s.tot_* is the area of all longitudinal reinforcement in the section of the element;

*φ* is the coefficient taken for a long-term load according to Table 6.2 from [51] depending on the flexibility of the element.

**Figure 5:**
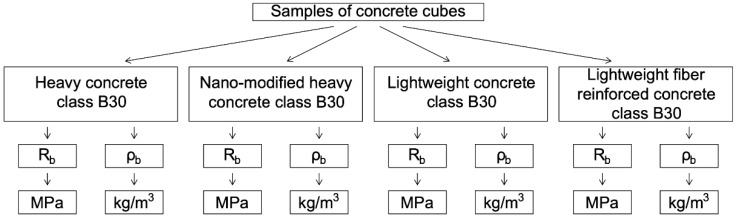
Plan of the physical experiment.

The plan of the physical experiment is shown in Figure 5.

For particle size analysis of micro silica, a Microsizer 201C laser particle analyzer (OOO VA Insatalt, St. Petersburg, Russia) was used in [43].

An X-ray phase analysis (XPA) of micro silica was carried out on an X-ray diffractometer HZG-4C (Freiberger Prazisionmechanik, Berlin, Germany).

The concrete mixture was made in a laboratory concrete mixer BL-10 (ZZBO LLC, Zlatoust, Russia). First, the dry components were mixed for 60 s, then the mixture was mixed with water and mixed until a homogeneous consistency was obtained. The fiber-reinforced concrete mixture was prepared according to the algorithm described in [29]. In the manufacturing of nanomodified heavy concrete, in order to increase the homogeneity of the binder (a mixture of cement and micro silica), the mixture of powders was processed in an Activator-4M homogenizer (OOO Chemical Engineering Plant, Novosibirsk, Russia). Furthermore, the modified binder, together with other dry concrete components—sand and crushed stone—was mixed in a concrete mixer for 60 s. After that, the resulting mixture of dry components was mixed with water, and mixed until a homogeneous consistency was obtained. Then, cube samples were formed with a rib size of 100 mm, and the mixture was compacted in molds on a laboratory vibration platform SMZh-539-220A (OOO IMASH, Armavir, Russia). After that, concrete cube samples were placed in a normal hardening chamber and stored for 28 days at a relative air humidity of at least 95% and a temperature of (20 ± 2) °C. Twenty-four hours after being manufactured, the samples were removed from the molds.

To obtain a lightweight fiber-reinforced concrete, some large and small dense aggregates were replaced by a porous aggregate—slag pumice—the fractions and dosages of which were taken in accordance with Tables 8 and 9 (composition S4) from [46].

The parameters of the composition of the concrete mix were applied similarly to Table 7 from [46].

The composition of heavy concrete nanomodified with micro silica was chosen according to the recommendations [43].

Compressive strength tests of specimens were carried out in accordance with GOST 10180 “Concretes. Methods for strength were determined using reference specimens” [53] on an IP-1000 hydraulic press (OOO NPK TEHMASH, Neftekamsk, Russia). All samples of the same series were tested at the age of 28 days for no more than 1 h. The samples were loaded continuously at a constant rate of load increase until failure. In this case, the loading time of the sample until its destruction was at least 30 s. The maximum force achieved during the test was taken as the breaking load. Sample cubes were installed on one of the selected faces on the lower support plate of the press which was centrally relative to its longitudinal axis, using the marks applied to the press plate. After placing the sample on the press support plate, the upper press plate was aligned with the upper support face of the sample so that their planes completely adjoined to one another. The sample was loaded to failure at a constant rate of load increase (0.6 ± 0.2) MPa/s. The compressive strength of concrete was calculated with an accuracy of 0.1 MPa using the formula:

(2) $$R=\alpha \frac{F}{A}$$

where *F* is the breaking load, N;

*A* is the area of the working section of the sample, mm^2^;

and α is a scale factor for converting the strength of concrete to the strength of concrete in samples of a basic size and shape (for cubes with an edge size of 100 mm, it is 0.95).

The strength of concrete in a series of samples was determined as the arithmetic mean of the strength of the tested samples in a series of six samples—four samples with the highest strength.

The average density of samples in a state of normal humidity was determined according to GOST 12730.1 “Concretes. Methods of determination of density” [54]. The volume of the samples was calculated from their geometric dimensions. The sample sizes were determined with a caliper with an error of no more than 1%. The mass of samples is determined by weighing an error of no more than 0.1%. The average concrete density of each sample in the series was calculated with an error of up to 1 kg/m^3^ using the formula:

(3) $$\rho =\frac{m}{V}$$

where *m* is the mass of the sample, g;

*V* is the sample volume, cm^3^.

The average density of concrete was calculated as the arithmetic mean of the test results for all samples of the series.

In total, 2 series of sample cubes of each type of concrete were made and tested (in one series—6 sample cubes). That is, a total of 48 sample cubes of four types of concrete were tested (12 of each type).

The microstructure of fiber-reinforced samples was studied using a ZEISS CrossBeam 340 microscope equipped with an Oxford Instruments X-Max 80 X-ray microanalyzer (Carl Zeiss Microscopy GmbH Factory, Jena, Germany) [29,55].

The microstructure of micro silica-modified samples was studied using a VEGA II LMU microscope (Tescan, Brno, Czech Republic) [43,44].

## 3. Results

**Figure 6:**
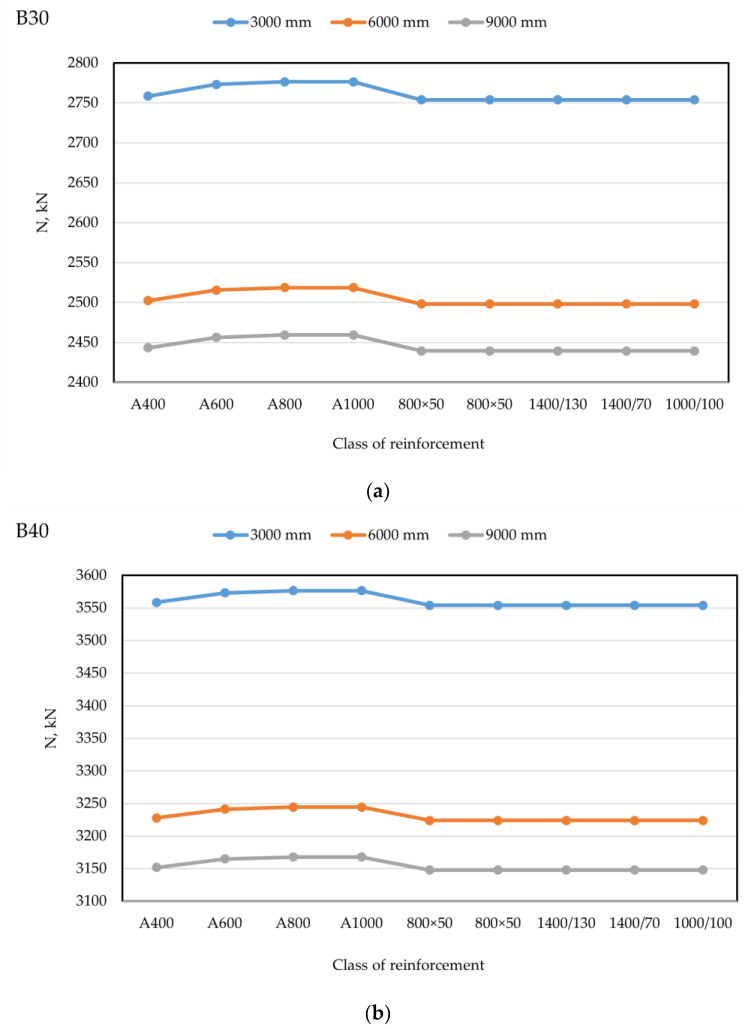
Dependence of the bearing capacity of reinforced columns of various lengths on the type and class of reinforcement when using concrete of class (**a**) B30; and (**b**) B40.

The results of a numerical experiment—calculation of the bearing capacity (limiting value of the longitudinal force N) of reinforced concrete columns with a cross section of 400 mm × 400 mm with a reinforcing bar diameter of 6 mm—are presented in Table 11 and Figure 6. The experiment varied the class of concrete, the length of the product, the type, and reinforcement class.

From the data of the numerical experiment, the results of which are presented in Table 11 and Figure 6, it can be seen that:

(1) the use of polymer composite reinforcement instead of steel, leads to an insignificant decrease (by 0.5–1.0%) in the bearing capacity of columns operating in compression;

(2) the bearing capacity of elements in the form of columns working in compression does not depend on the type of polymer composite reinforcement used in the study;

(3) the difference in bearing capacity between 3000 mm and 6000 mm columns is 9–10%, and between 6000 mm and 9000 mm columns it is 2% to 3%, regardless of other parameters considered;

(4) increasing the concrete grade from B30 to B40 results in an increase in the bearing capacity of compressive columns by up to 30%.

After analyzing the data obtained as a result of the numerical experiment in Figure 6, we will explain the resulting difference in the data obtained with different initial parameters. Moreover, polymer composite reinforcement leads to an insignificant decrease in the bearing capacity due to the high strength characteristics of most modern polymer composite reinforcements, that is, the high strength characteristics of polymer composite reinforcement, with all its unambiguous advantages, primarily that it is lightweight, also allows it to maintain the bearing capacity of the entire structure at a high level. As for the lack of dependence of indicators on the type of polymer composite reinforcement, this can be explained by the fact that the polymer composite rods used in the study are modern, have passed various stages of testing, and have high strength characteristics, regardless of their type. Some decreases in the difference of the bearing capacity between the columns depending on the length can be explained, among other things, by a change in the geometric parameters and the working design scheme when the structure is loaded. Undoubtedly, the most important parameter for compressible reinforced elements using heavy concrete is the concrete class, which is reflected as a result of a numerical experiments, namely, with an increase in the class, which we achieve using our developed methods. Moreover, the bearing capacity of the columns also increases, and significantly—up to 30 percent. All this allows us to talk about the effectiveness of the proposed integrated approach.

**Figure 7:**
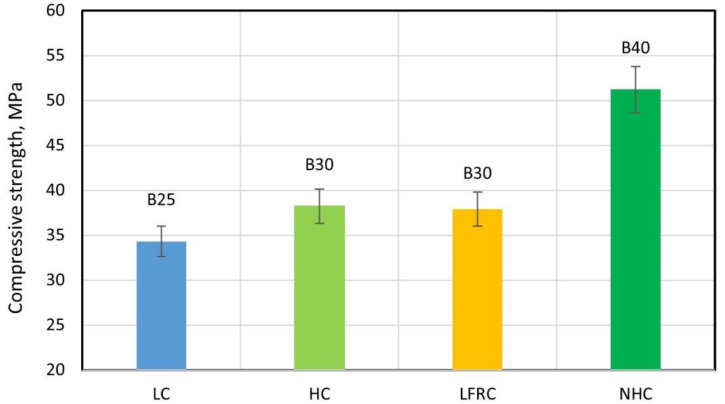
Compressive strength of the studied types of concrete.

The results of the numerical experiment are presented in Table 12 and Figure 7.

**Figure 8:**
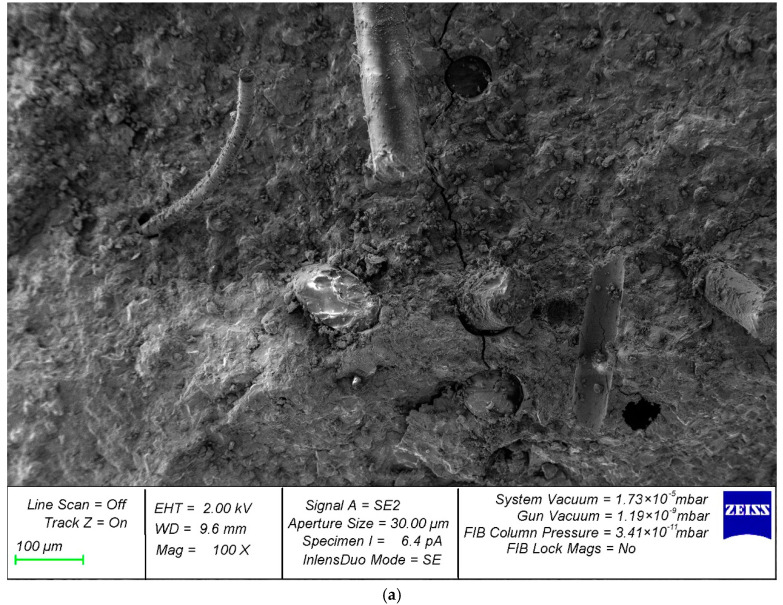

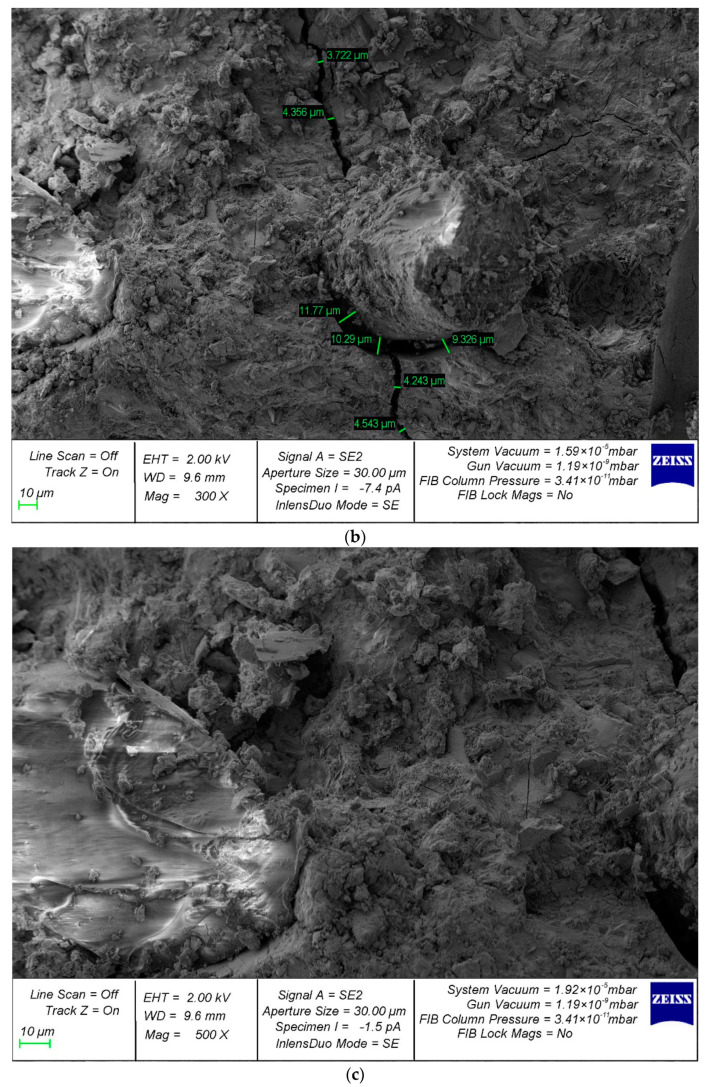

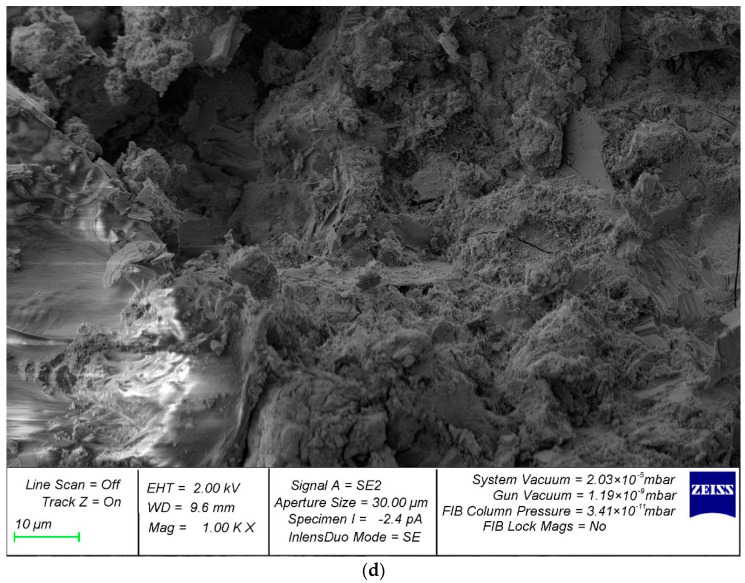
Photographs of the microstructure of a fiber-reinforced sample with magnification: (**a**) 100×; (**b**) 300×; (**c**) 500×; and (**d**) 1000×.

Figure 8 shows photographs of the microstructure of cement stone reinforced with glass fibers.

From the data of the physical experiment, the results of which are presented in Table 12 and Figure 7, it can be seen that:

(1) the difference between lightweight and heavy concrete is up to 10% (concrete grade reduction from B30 to B25);

(2) glass fiber reinforcement brings the compressive strength of lightweight concrete up to that of heavy concrete (upgrading concrete from B25 to B30);

(3) nano-modification of heavy concrete with micro silica resulted in an increase in the compressive strength of concrete up to 35%, whereas the concrete grade increased from B30 to B40. 

Photographs of the microstructure of the fiber-reinforced sample are shown in Figure 8.

The results of microscopic studies with high-resolution photographs and high magnification made it possible to determine the physical nature and mechanism of the destruction of fiber-reinforced composites, which are the basis for building elements. The microphotographs (Figure 8a–d) clearly show that the nature of crack development directly depends on the rational distribution of fibers in the body of the cement matrix. In areas that make it possible to evaluate the usefulness of a rational distribution of fibers (Figure 8a), there are no microcracks in the region of the fibers (Figure 8c,d); however, with improper homogenization and distribution of the reinforcing fiber, in the area of the resulting fiber bundles, defects appear at the same time, expressed in microcracks (Figure 8b), which, in principle, correlates with the analogue of this phenomenon at the macro level—excessive density of reinforcement of complex structures. Thus, microscopic studies confirm the thesis not only with regard to the initial characteristics and quality of the fibers used, but also the importance of their distribution and homogenization in the body of the matrix, since microcracking already occurs at this stage and can develop at the macro level.

**Figure 9:**
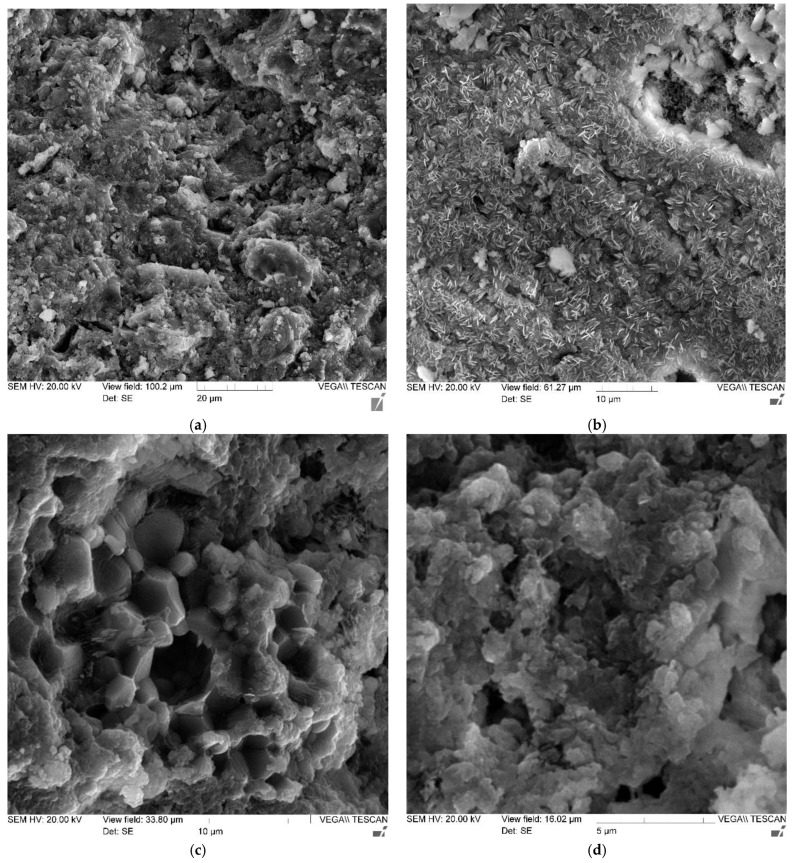
Photographs of the microstructure of micro silica-modified samples with magnification: (**a**) 1000×; (**b**) 2000×; (**c**) 3000×; (**d**) 7000×.

Figure 9 shows photographs of the microstructure of cement stone modified with micro silica.

Figure 9a–d show that micro silica nanomodification [43,44] forms a denser and more uniform structure that looks ordered and connected. This is due to the formation of a structure mainly from low-basic calcium hydrosilicates with nuclei of crystalline phases and local accumulations of portlandite, as well as a decrease in recrystallization activity after a period of accelerated hydration and an increase in the degree of cement hydration. Photographs of the microstructure of samples nanomodified with micro silica confirm the increase in the compressive strength of concrete that was obtained by us in experimental studies, and accordingly, its bearing capacity.

The description and analysis of photographs of the microstructure of samples nanomodified with micro silica were carried out, among other things, in accordance with [43,44].

## 4. Discussion

The compared criteria for determining the role and place of research in modern science included such parameters as the size of the section of products, the class of concrete, the length of the product, the types of reinforcement, the class of reinforcement, and the diameter of the rod, which ultimately influenced the limiting value of the longitudinal force for compressible elements.

Thus, according to the indicated criteria, we evaluated the effectiveness of each of the considered methods when comparing different types of materials:

- in relation to nanomodified concrete;
- in relation to lightweight fiber-reinforced concrete;
- in relation to lightweight concrete;
- in relation to control heavy concrete.

In terms of the result achieved, we note that the most effective compositions obtained showed strength gains from 10% to 34%. Ultimately, this led to an increase in bearing capacity of up to 30%.

It is possible to explain the obtained quantitative increases in concrete indicators by the observed qualitative picture of these changes. Thus, we have obtained significant improvements, which, in an integrated approach, led to an improvement in the structure of concrete, a decrease in the weight of concrete and, thereby, loads on the base. At the same time, the bearing capacity due to a rationally selected recipe and technological factors continues to remain at a high level. Thus, we have achieved a significant improvement in the qualitative picture, which leads to a synergistic effect from the developed proposals. This ultimately leads to an improvement in the characteristics of materials, then to an increase in the bearing capacity, and ultimately, to greater economic efficiency of the created building structures.

This is in agreement with the results of the authors who dealt with the previously mentioned issue [2,3,10,16,23,28]; however, unlike [3], where an increase in the bearing capacity of columns up to 35% was provided by high-strength steel, in our study we achieved a comparable effect, but by using much lighter and more ergonomic reinforcing rods in combination with one of the proposed recipe solutions. In addition, unlike [18,36], in our study, an exceptionally positive effect was observed without a decrease in the compressive strength of concrete and reinforced concrete. At the same time, in the case of lightweight fiber-reinforced concrete, we achieve greater versatility of the resulting concrete and elements by increasing the deformability, thus making the fracture pattern more viscous due to additional fiber reinforcement and causing the appearance of some damping effect, due to the combination of fiber and porous filler.

In the case of concrete being nanomodified with micro silica and a reinforced element, we obtain a cheaper element with improved characteristics due to the use of industrial waste [37]. This leads to a reduction in waste and allows for more environmentally friendly and more economical construction, which, in general, will lead to a reduction in the cost of construction by up to 10–12%, according to preliminary estimates of industrial partners.

Numerical experiments have established that one of the most important criteria in the calculation of elements is the class of concrete. At the same time, this characteristic has an effect for all types of reinforcement and all types of reinforcing bar material, for various section sizes, and bar lengths. Due to this, the compressible elements are significantly dependent on the class of concrete used.

Therefore, we applied such a numerical physical approach when conducting experiments to produce a direct correlation between these quantities and to prove the effectiveness and possibility of obtaining reinforced building elements based on concrete using various types of reinforcing bars with different densities and weights.

It has been confirmed that by increasing the class of concrete, it is possible to increase the bearing capacity of the element, or by reducing the mass of the element, it is possible to maintain the bearing capacity at the same level. All of this became possible thanks to the use of various recipe-technological methods at the stage of manufacturing a concreted reinforced structure, which works as a compressed element.

Thus, due to inexpensive recipe methods, namely, nanomodification with the help of production waste, or the use of lightweight dispersed-reinforced concrete to obtain such structures, we achieve significant technological, design, and installation effects. The design effect reduces the importance of the rod reinforcement and supplements the improved element with dispersed fiber reinforcement. The technological effect is achieved using concrete modification, a more rational selection of its formulation, and by improving the characteristics of the concrete, an increased bearing capacity of elements arises. The construction and installation effect lies in the fact that such lightweight universal structures are more applicable and have a wider area of operation at construction sites of various levels of responsibility and different directions.

The proposed integrated system approach has a number of advantages over existing approaches. In particular, our developed approach makes it possible to take into account both the characteristics of the materials used and the quality of the technological process of concrete production, that is, technological and design advantages, expressed in design and design efficiency, using software, and taking into account the characteristics of materials, especially in relation to lightweight structures of new types. Such structures are designed for the difficult conditions of modern construction and certainly require an integrated systematic approach in their creation, calculation, and design.

## 5. Conclusions

A new integrated system engineering approach to the design of building structures is proposed, which involves minimizing their cost and weight through numerical simulations and experimental verificationss of the operation of reinforcing bars made of various materials in concretes of various densities.

It has been confirmed that by increasing the class of concrete, it is possible to increase the bearing capacity of the element, or by reducing the mass of the element, it is possible to maintain the bearing capacity at the same level.

The control of the bearing capacity of reinforced building structures, using the example of compressed elements, is proposed to be carried out by the developed recipe-technological methods at the manufacturing stage.

The economic and environmental efficiency of the proposed engineering methods, namely, nanomodification with the help of production waste or the use of lightweight dispersed–reinforced concrete to obtain such structures, has been revealed. This allows significant effects to be achieved, such as:

- design effect, which consists of reducing the importance of rod reinforcement and supplementing the improved element with dispersed fiber reinforcement;
- technological effect, which is achieved through the use of concrete modification, a more rational selection of its formulation, and by improving the characteristics of a concrete by obtaining an increased bearing capacity of elements from it;
- construction and installation effect, which consists of the fact that such lightweight universal structures are more applicable and have a wider area of operation at construction sites of various levels of responsibility and various directions.

The most effective concrete formulations have demonstrated strength gains ranging from 10% to 34%. Ultimately, this led to an increase in the bearing capacity of the elements up to 30%.

The use of such an integrated lean approach will save up to 20% of resources during construction.

Future prospects, and ways to develop the study further, are planned in terms of bending elements with various types of concrete and reinforcement. Moreover, taking into account the improvement of the technology for calculating and designing lightweight building structures, it is of interest to develop the results obtained in the direction of concrete with an improved structure. Thus, in further research, we will apply the developed integrated system approach for concretes obtained using technologies other than standard vibration.

## Figures and Tables

**Table 1:** Physical and mechanical characteristics of Portland cement.

Density, kg/m^3^	Normal Cosistency, %	Blaine Specific Surface Area, cm^2^/g	Setting Time, min		Compressive Strength at 28 Days, MPa	Bending Strength at 28 Days, MPa
			Start	End		
3120	24.8	3586	140	260	55.3	6.95

**Table 2:** Chemical composition of Portland cement.

Cement Title	Oxid Content, %											L.O.I.	Cl
	SiO_2_	Al_2_O_3_	CaO	Fe_2_O_3_	MgO	TiO_2_	P_2_O_5_	SO_3_	Na_2_O	K_2_O	Na_2_O_equiv._		
Additive-free Portland cement CEM 0 52.5N GOST 31108-2020	21.1	4.9	62.7	4.4	1.7	0.1	0.1	2.8	0.3	0.6	0.57	0.7	0.03

**Table 3:** Mineralogical composition of Portland cement.

Cement Title	Mineral Content, %				
	C_3_S	C_2_S	C_3_A	C_4_AF	CaO_fr._
Additive-free Portland cement CEM 0 52.5N GOST 31108-2020	75.5	8.1	4.5	11.4	0.5

**Table 4:** Physical and mechanical characteristics of crushed granite.

Fraction	Bulk Density, kg/m^3^	True Density, kg/m^3^	Crushing, wt %	Lamellar Grain Content and Needle-Shaped Forms, wt. %	Void Index, %
5–20	1420	2640	10.8	7.5	43

**Table 5:** Physical characteristics of dense fine aggregate.

Fineness Modulus	Content of Dust and Clay Particles, %	True Density, kg/m^3^	Bulk Density, kg/m^3^	Clay Content in Lumps, %
1.72	1.4	2665	1422	0.1

**Table 6:** Characteristics of the steel reinforcement.

Characteristics	Steel A400	Steel A600	Steel A800	Steel A1000
Yield strength, MPa	380	570	760	970
Tensile strength, MPa	580	860	1010	1220
Modulus of elasticity, GPa	200			
Elongation, %	16	9	7	6
Density, t/m^3^	7.0	7.2	7.4	7.5

Note: Steel A400—steel reinforcement class A400; Steel A600—steel reinforcement class A600; Steel A800—steel reinforcement class A800; Steel A1000—steel reinforcement class A1000.

**Table 7:** Characteristics of the used polymer composite reinforcement.

Indicator Title	GCR	BCR	CaCR	ACR	CoCR
Tensile strength, MPa	800	800	1400	1400	1000
Tensile modulus, GPa, not less than	50	50	130	70	100
Ultimate compressive strength, MPa, not less than	300	300	300	300	300
Ultimate strength at cross section, MPa, not less than	150	150	350	190	190

Note: GCR—glass composite reinforcement—a polymer composite containing a continuous reinforcing fiberglass filler; BCR—basalt composite reinforcement—a polymer composite containing a continuous reinforcing filler made of basalt fiber; CaCR—carbon composite reinforcement—a polymer composite containing a continuous carbon fiber reinforcing filler; ACR—aramid composite reinforcement—a polymer composite containing a continuous reinforcing filler of aramid fiber; CoCR—combined composite reinforcement—glass composite or basalt composite, or carbon composite, or aramid composite, additionally filled with a continuous reinforcing filler from another type or types of fiber [45].

**Table 8:** Chemical composition of micro silica.

Title	Oxid Content, %							
	SiO_2_	Al_2_O_3_	Fe_2_O_3_	CaO	MgO	R_2_O	SO_3_	L.O.I.
MS-85	81.9	1.5	2.8	1.2	0.3	0.9	3.8	7.2

**Table 9:** Physical and mechanical characteristics of glass fiber.

Density, g/cm^3^	Tensile Strength, GPa	Elastic Modulus, GPa	Fiber Length, mm	Elongation, %
2.6	1.8	70	12	1.5

**Table 10:** Physical and mechanical characteristics of slag pumice.

Fraction, mm	Bulk Density, kg/m^3^	True Density, kg/m^3^	Strength, MPa	Void, %
5–10	608	1320	0.8	52
1.25–2.5	727	1408	-	54

**Table 11:** Results of calculating the limiting value of the longitudinal force (bearing capacity).

Product Section SIZE, mm × mm	Concrete Class	Product Length, mm	Reinforcement Type	Reinforcement Class	Rod Diameter, mm	Ultimate Value of Longitudinal Force *N*, kN
400 × 400	B30	3000	steel	A400	6	2758.4
				A600		2773.1
				A800		2776.5
				A1000		2776.5
			GCR	800 × 50		2753.9
			BCR	800 × 50		2753.9
			CaCR	1400/130		2753.9
			ACR	1400/70		2753.9
			CoCR	1000/100		2753.9
	B40		steel	A400		3558.4
				A600		3573.1
				A800		3576.5
				A1000		3576.5
			GCR	800 × 50		3553.9
			BCR	800 × 50		3553.9
			CaCR	1400/130		3553.9
			ACR	1400/70		3553.9
			CoCR	1000/100		3553.9
	B30	6000	steel	A400		2502.2
				A600		2515.6
				A800		2518.7
				A1000		2518.7
			GCR	800 × 50		2498.2
			BCR	800 × 50		2498.2
			CaCR	1400/130		2498.2
			ACR	1400/70		2498.2
			CoCR	1000/100		2498.2
	B40		steel	A400		3228.0
				A600		3241.3
				A800		3244.4
				A1000		3244.4
			GCR	800 × 50		3223.9
			BCR	800 × 50		3223.9
			CaCR	1400/130		3223.9
			ACR	1400/70		3223.9
			CoCR	1000/100		3223.9
	B30	9000	steel	A400		2443.2
				A600		2456.2
				A800		2459.2
				A1000		2459.2
			GCR	800 × 50		2439.2
			BCR	800 × 50		2439.2
			CaCR	1400/130		2439.2
			ACR	1400/70		2439.2
			CoCR	1000/100		2439.2
	B40		steel	A400		3151.8
				A600		3164.8
				A800		3167.8
				A1000		3167.8
			GCR	800 × 50		3147.8
			BCR	800 × 50		3147.8
			CaCR	1400/130		3147.8
			ACR	1400/70		3147.8
			CoCR	1000/100		3147.8

**Table 12:** Physical and mechanical parameters of the studied types of concrete.

Type of Concrete	Average Density, kg/m^3^	Compressive Strength, MPa
Lightweight concrete	1880	34.3 ± 1.7
Heavy concrete (control)	2340	38.2 ± 1.9
Lightweight fiber concrete	1890	37.9 ± 1.9
Nano-modified heavy concrete	2430	51.2 ± 2.5

## Data Availability

The study did not report any data.
